# Potential of Halophytes as Sustainable Fodder Production by Using Saline Resources: A Review of Current Knowledge and Future Directions

**DOI:** 10.3390/plants12112150

**Published:** 2023-05-29

**Authors:** Maria Hasnain, Zainul Abideen, Faraz Ali, Mirza Hasanuzzaman, Ali El-Keblawy

**Affiliations:** 1 Department of Biotechnology, Lahore College for Women University, Lahore 35200, Pakistan; 2 Dr. Muhammad Ajmal Khan Institute of Sustainable Halophyte Utilization, University of Karachi, Karachi 75270, Pakistan; 3 School of Engineering and Technology, Central Queensland University, Sydney 40939, Australia; faraz_rao@yahoo.com; 4 Department of Agronomy, Faculty of Agriculture, Sher-e-Bangla Agricultural University, Dhaka 1207, Bangladesh; 5 Kyung Hee University, 26 Kyungheedae-ro, Dongdaemun-gu, Seoul 02447, Republic of Korea; 6 Department of Applied Biology, College of Sciences, University of Sharjah, Sharjah P.O. Box 2727, United Arab Emirates

**Keywords:** biosaline agriculture, fodder, salt tolerance, toxins, feed, phytoremediation

## Abstract

Good quality water and arable land are required for both domestic and agricultural uses. Increasing population leads to urbanization and industrialization increasing the need to share these resources and creating threats to the food supply. Higher meat consumption requires mitigation strategies to protect food and mitigate economic crises, especially in developing nations. The production of food crops for energy purposes and lower yield due to climate change increase food prices as well as have a negative impact on the economy. Thus, an alternative food source is required featuring high forage components to reduce grazing periods and to prevent rangeland degradation. Halophytes can tolerate high salinity and can be easily grown for fodder in coastal areas where fodder is a problem. Varied climate conditions offer opportunities to grow suitable halophytes for specific purposes. One important feature is their use as fodder. To reduce food shortages, saline areas could be used to grow nutritive and productive halophytic forage. Wild plants have undesirable metabolites produced in harsh conditions which may be harmful for ruminant health. Halophytes have moderate amounts of these metabolites which are nontoxic. Halophytes can be grown without intruding on agricultural lands and freshwater resources and could promote livestock production which may improve the socio-economic conditions of poor farmers in a sustainable and ecofriendly manner.

## 1. Introduction

Agricultural land is diminishing continuously due to climate change, increasing population pressure, and adverse environmental conditions [1]. About 831 million hectares of the cultivated area is affected by soil salinity, which includes 397 million hectares, while the remaining 434 million hectares are sodic soils. Among the irrigated land, 20% is affected by salinity which accounts for approximately 45 million hectares. Approximately 1.5 million hectares of land is ruined because of salinity [2]. It has been estimated that at least three hectors of arable land are lost every minute in the world due to salinity. If it continues in such a way, then 50% of the arable lands will be depreciated through the twenty-first century [3]. There are many factors that cause salinity, such as the rise of the water table, irrigation with sea water, and poor drainage [4]. Salinity causes adverse impacts on plant germination, growth, reproducibility, photosynthesis, respiration, transpiration, and metabolic processes, and ultimately leads to plant death under extreme saline conditions. Salinity is a very complex or continuous global problem which is difficult to remediate and requires a multidisciplinary approach which is cost and labor extensive [5]. There are many ways for the better utilization of saline land, including a number of agronomic approaches, phytoremediation, and harvesting salt tolerate crops. Many agronomic approaches and phytoremediation are very cost-effective for remediation. Exploring new harsh environment tolerating plant species as a substitute to conventional animal feed is important, as is and facing all the challenges of population pressure on insufficient food assets. The increasing demand for animal feed enacts pressures. As natural resources are not utilized in developing countries, further attention is required for economical feeding by using non-conventional feed resources while sustaining animal production at optimum levels [6]. Thus, halophytes could play an important role in the well-being of different people.

**Figure 1:**
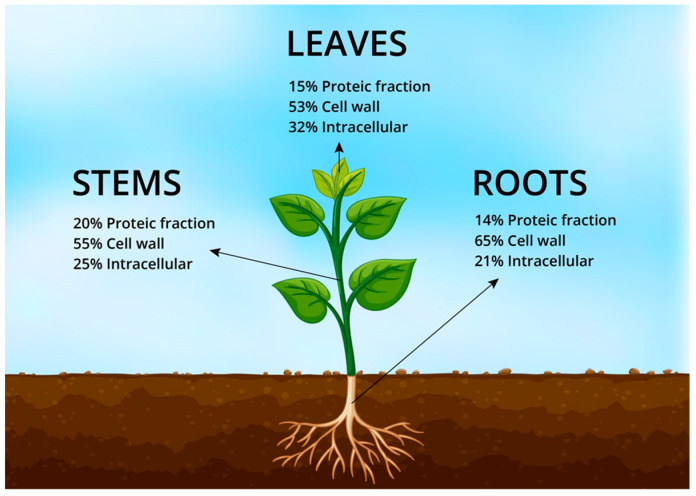
Proteic fraction of halophyte *Atriplex* species in cell wall and intracellular fraction in leaf, stem, and root compositions.

Around 1% of the world’s flora constitutes halophytes which have the ability to grow or reproduce in more than 200 mM of NaCl-containing environment. Halophytes have exceptional morphological and anatomical features to cope with the saline environment [7]. Halophytes improve salt soil with their physiological processes, such as ion compartmentalization, salt inclusion, salt excretion, ion transportation, and antioxidant and osmotic regulation. Halophytes can be utilized for industrial, ecological, and agricultural tenacities [8]. Halophytes have the potential to fulfill all the basic needs of a growing population with their numerous commercial norms such as foodstuff, forage, energy, medications, and revegetation in third-world countries. Within grazing schemes, halophytic fodders serve as drought reserves to plug annual feed shortages [9]. In Europe, due to the nutritional value (Figure 1) of *Atriplex* species, they are used to fill the seasonal feed gap, or *S. bigelovii* is used as a protein complement. Halophytes have the potential to provide higher yields in a saline environment than other conventional crops, while their yield potential depends on the plant species and the salt quantity [10].

## 2. Feeding and Nutritive Value

Feeding value is the function of voluntary feed intake and the nutritive value of biomass which impacts meat, milk, and wool production. To meet nutritional requirements in the grazing system, it is important to deliberate the availability of halophytes and non-halophytes to provide complementary forages [11]. Supplements implicate the economic, labor, and transport costs. Therefore, to maximize the dependence on additional feed, we should maximize the feeding value of halophytes. Nutritive value is the function of digestibility of the nutrients and the efficiency of the nutrients used for animal production. Crude protein, minerals, and metabolites are the major contributors to nutritive value [12].

### 2.1. Metabolizable Energy

The function of the digestible organic matter in dry matter (DM) is known as metabolizable energy. This is generally lower in halophytes than in non-halophytes because of the presence of less organic matter [13]. *D. spicata* and *S. virginicus* provide a diet to animals with supplementary energy concentration. Researchers have studied the relationship between salinity and fiber value. In *C. dactylon,* neutral detergent fiber increased by 5%, while in *T. ponticum* it decreased by 3% [14]. Attia-Ismail [15] found no relationship between salinity and fiber content among five different halophytic species, *A. lagopoides*, *S. tremulus*, *P. paspaloides*, *P. geminatum*, *A. nummularia,* and *S. tragus,* which have 2.30, 2.38, 2.53, 2.33, 2.82, and 2.56 M cal kg^−1^ energy, respectively.

### 2.2. Protein and Nitrogen

Ruminants require the minutest protein for their growth. Adult ruminants require approximately 7–9% protein, while growing or lactating ruminants required 14–18%. Protein is degraded by the rumen microbe, but some of the degraded protein is converted back into microbial protein by the rumen microbe and passes down the gastrointestinal tract for amino acid absorption [16]. Undegraded dietary protein resists the rumen microbe and is absorbed in the lower gastrointestinal tract. Halophytes with protein content are listed in Table 1. Low protein content in halophytes could be enhanced by agronomic practices, for example, by harvesting halophytic grasses in the presence of nitrogen (N), fertilizer, and seawater [15]. In *S. virginicus,* protein content increased from 6.8–9.0% when irrigated with 12.5–50% seawater [17]. The relationship between protein content and soil salinity is not consistent, for example, protein content was 12% in *C. gayana* and 16% in *C. dactylon*, irrespective of salinity [18]. *M. alba* achieved 13% protein from using Rhizobia in root nodules to fix N [19].

In ruminants, the N compounds glycinebetaine (GB) and proline (Pro) have both positive and negative effects. GB acts as a methyl doner in protein for the recycling of amino acids and energy metabolism which is important in ruminants’ muscle growth. GB also assists in choline production, improves lean and fat ratio in meat, and also improves carcass composition [20]. In ruminants’ diets, more than 50% GB is degraded by rumen microbes. Pro as hydroxy proline is associated with collagen, which can be absorbed directly into the small intestine. Ruminants have the ability to synthesize adequate Pro to meet their necessities as it is important for their growth and production [21].

**Table 1:** List of halophytes with nitrogen and crude protein contents on % dry biomass.

Halophytes	% Nitrogen	% Protein	References
*A. saligna*	2	14	[17]
*A. brevifolia*	1.6	9	[22]
*A. halimus*	2	13	
*A. nummularia*	2	13	
*A. repanda*	3	21	[23]
*H. ammodendron*	2	14	
*H. salicornicum*	2	17	[24]
*H. strobilaceum*	1	7	
*K. foliatum*	3	19	[25]
*K. caspica*	2	12	
*L. chilense*	2	13	
*P. communis*	2	12	[26]
*S. tetranda*	1	7	
*R. soongorica*	1	10	[7]
*S. foliosa*	3	17	
*S. fruticosa*	2	12	
*T. mannifera*	1	7	[18]
*T. crinita*	1	10	
*Z. album*	1	6	

### 2.3. Sulfur

Plants have sulfur (S) concentrations mainly ranging from 0.05–0.5% DM. Sulfur is the main component of several vitamins, insulin, coenzyme A, and three amino acids (cystine, cysteine, and methionine). These amino acids are essential for protein synthesis. 0.2% DM S is recommended in the diet of sheep and 0.15% DM in the diet of cattle [25]. Sulfur is used with N. The optimal ratio of N:S is 12.5:1 which is considered best for sheep. Sulfur causes toxicity when converted into sulfide in the rumens of animals instead of ruminal protein. Sulfides reduce the reduced copper (Cu) absorption, which induces Cu deficiency. Sulfides reduce rumen motility and decrease voluntary feed intake [27].

### 2.4. Minerals

Halophytes are different from other plants due to their ability to make osmotic adjustments, which in *C. quinoa* affects the mineral contents of the edible plants. Salinity tolerance is achieved by sodium (Na) and chloride ion exclusion from the root surface and ion secretion from the leaves. The total ash from halophytic *C. quinoa* contains 63–81% Na, potassium (K), and chloride ions, whereas legume chenopods contain only 40% [28]. Under salt stress, halophytes have the ability to modify their salt glands to excrete excess ions from inside the plant body, which also impacts the salt concentration in halophytic feed consumed by ruminants [22]. Many halophytes thicken their leaves in salt stress which increases tissue hydration. *A. lentiformis* had 2.4 g g^−1^ OM water content and 15.9% DM ash concentration, while *S. europaea* had 23.7 g g^−1^ OM water content and 51.4% DM ash content [29]. Consumption of salt accumulating shrubs, such as *Atriplex* species, can cause toxicities in grazing ruminants by the accumulation of S and selenium [30]. Sheep were allowed to adapt to feed for three weeks, but over a consequent week, the sheep had net losses of magnesium 0.8, calcium (Ca) 0.6, and K 0.4 g per day^−1^. These facts show that *Atriplex* sp. as a solitary feed are inappropriate for ruminants. Hence, an advanced study is a prerequisite to evaluating the mineral stability in animals [30].

### 2.5. Organic Acids

For osmotic adjustment, halophytes use organic acids such as divalent anion oxalate, malate, and trivalent citrate, as well as anions, to achieve cation–anion balance. Centofanti and Bañuelos [24] studied twenty-one halophytes and concluded that five species from Chenopodiaceae and one from Caryophyllaceae had oxalate of more than 50 mM, 26–62% of the total anionic charge, and 5% DM. One specie from Brassicaceae had more than 70 mM citrate with 21% of the total anionic charge and about 15% DM [16]. Leaves of *Atriplex* sp. can produce 3.6–6.6% DM and about 40% total between cation and anions. In *A. spongiosa*, 76% of extra cations were stable by oxalate. Oxalic acid forms insoluble calcium oxalate, which reduces Ca concentrations in the blood of animals causing milk fever and problems in bone development [31]. Moreover, the sleet of calcium oxalate in the kidneys leads to kidney destruction. Oxalate has the ability to bind to other minerals such as iron (Fe), manganese (Mn), zinc (Zn), and Cu [32]. In the leaves of *A. spongiosa,* oxalate binds to all present Ca^2+^. Therefore, oxalate is the cause of the loss of Ca in sheep grazing. Feed containing Ca supplement for animals is a significant tool to improve the utilization of halophytes [33].

### 2.6. Antioxidants

In the ruminant diet, tocopherol, or vitamin E is a very prevailing antioxidant. Tocopherol is present in the thylakoid membranes of chloroplasts which defend lipids from oxidation by ROS [34]. The concentration of α-tocopherol varies according to different ecological stresses as well as during different growth stages of plants. A deficiency of tocopherol can be the reason for nutritional myopathy and animal death. *Atriplex* sp. contains 116–139 mg kg^−1^ DM α-tocopherol [30]. Vitamin E present in *Atriplex* sp. suspension influences the oxidative transformation of oxymyoglobin to brown metmyoglobin by oxidation of lipids in meat, which improves the flavor as well as the shelf-life of meat. Vitamin A is another antioxidant present in halophytic shrubs [35]. *A. nummularia* contains 41 mg kg^−1^ dry mater vitamin A. In ruminants, vitamin A improves visualization, immunity, bone development, and heart disorders. During droughts, the threat of vitamin insufficiency in ruminants is relatively high due to the lack of access to green feed [36].

## 3. Halophytes and Secondary Metabolites

**Figure 2:**
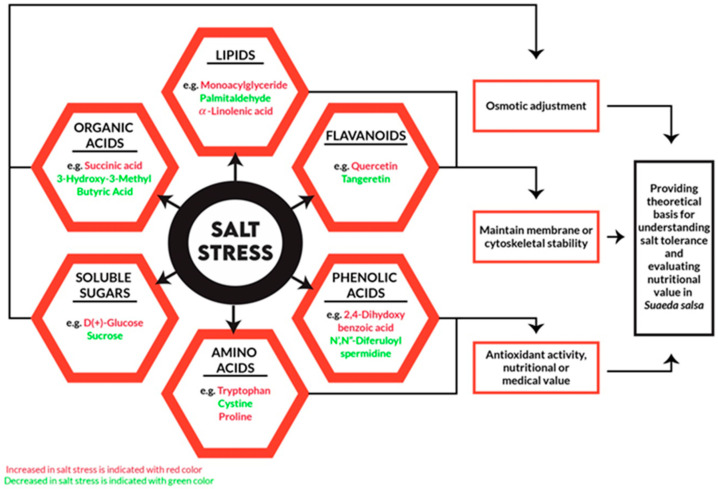
Synthesis of secondary metabolites in halophyte under salt stress.

Plant secondary metabolites are small molecular weight metabolically produced compounds, also referred to as phytochemicals. Plants produce secondary metabolites for protective purposes from environmental stresses (Figure 2). Approximately 80,000 secondary metabolites are naturally present in plants [37]. Some of them are valuable, but many of them have no nutritive value and have harmful properties. Ruminants are more tolerant to toxic plants than other animals. There is a difference in tolerance even within ruminant species, their ages, or their physiological status [38]. Sheep are more resistant to plant secondary metabolites than cattle. Due to long exposure to toxic plants, some animals have the ability to develop resistance against them. Ruminants have adjusted to *H. glomeratus,* which is an oxalate-containing plant that is lethal to non-adapted animals [16].

### 3.1. Phenolic Compounds

Phenolic compounds comprise simple phenols to condensed tannins. Flavonoids are the low molecular weighted phenolic compounds found in all plants. Flavonoids have numerous healthful effects because of their anti-mutagenic and anti-carcinogenic properties (Table 2). Flavonoid supplements reduce lipid peroxidation in sheep, improve anti-oxidative status, and increase milk production [39]. After lignins, tannins are the subsequent most profuse plant metabolite. Tannins have the ability to form strong complexes with proteins which is their utmost imperative nutritive and toxicological feature (Table 3). They can also form campuses with carbohydrates and digestive enzymes in the feed. Less than 4% tannins promote protein escape from digestion which upturns the absorption of the amino acids, while higher amounts decrease feed ingestion which can be dangerous [40]. Ruminants are more tolerant to tannins and these have survival benefits such as escalations in wool and milk production as well as ovulation rate. Soluble dietary proteins cause bloat by stabilizing foam in the rumen which can trap gas bubbles [15]. Tannins prevent rumen bloating and anathematic effects. By reducing rumen methane production from organic matter, tannins utilize efficient energy to increase the intestinal digestibility [41].

### 3.2. Nitrates, Saponins, and Mimosine

Nitrates are naturally existing compounds in plants. Environmental conditions and plant species affect the nitrate concentration in the feed of livestock. Nitrates are non-toxic, but nonetheless their metabolites (NO_3_ and NO) are toxic. Rumen microbes convert nitrates to ammonia by integrating nitrates into microbial protein. Their toxicity is influenced by the level of nitrate intake in feed, whereby <1000 ppm is safe for pregnant animals and >4000 ppm is highly toxic [42]. Saponins is derived from *sapo*, a Latin word that means soap. In ancient times, saponin containing plants have been used for washing purposes. Plant seeds contain a saponins content of approximately 100 g kg^−1^, while more than 200 g kg^−1^ are found in press cake. About 0.10 g kg^−1^ saponins increase ruminants’ productivity but also cause gastrointestinal parasitism [16]. Mimosine is a secondary compound present in plants. It was first discovered in *M. pudica. L. leucocephala* leaves are rich in nutrients which make it a complete ruminates feed. Mimosine concentration in the leaves of *L. leucocephala* is 7.19% and in the seeds is 12.13% total protein dry weight. Mimosine comprises about 5% of the total protein in the plant [43]. Due to the toxicity of mimosine, *Leucaena* causes problems to ruminants: mainly, hair loss, infertility, and goiters. *S. jonesii* have the ability to overcome the mimosine toxicity problem by degrading mimosine. Supplementation of *Leucaena* diets with ferrous sulfate and moist heat treatment, the addition of iron salts, water washing or soaking of leucaena leaves, and development of new *Leucaena* hybrids are possible to elucidate the mimosine toxicity [44].

**Table 2:** Secondary metabolites composition of halophytes growing under saline lands and brackish water metabolites.

Halophytes	Flavonoids (%)	Nitrates (%)	Saponins (%)	Oxalates (%)	Phenols (%)	Net Tannins (%)	Condensed Tannins (%)	References
*A. stocksii*	0.705	0.659	0.589	0.841	-	-	-	[45]
*Acacia nilotica*	0.487	0.102	0.531	0.481	70	15	1	
*Toona cililate*					4	2.3	0.9	
*Avicennia marina*	0.604	0.567	4.638	1.621	16	-	-	[15]
*Chenopodium album*	0.281	0.321	0.447	0.631	-	-	-	[46]
*Salsola kali*	-	-	-	-	3	-	1.9	[47]
*Conocarpus erectus*	0.472	0.691	2.221	0.721	-	-	-	[48]
*Bauhinia variegate*	-	-	-	-	5	3.7	3.4	
*Convolvulus arvensis*	0.859	0.379	0.702	0.721	-	-	-	[22]
*Phoenix acaulis*	-	-	-	-	6	4.8	4.3	
*Cressa cretica*	0.572	0.393	0.665	0.721	9.5			
*Anogeissus latifolia*					17.5	16	0.4	[49]
*Enicostemma hyssopifolium*	0.991	0.392	1.351	1.441	-	-	-	[39]
*Carrisa spinarum*	-	-	-	-	6.6	4.5	4.6	
*Haloxylon stocksii*	0.427	0.345	2.112	2.341				[50]
*Ougenia oojeiuealis*	-	-	-	-	4.2	2.9	2.6	
*Heliotropium bacciferum*	0.455	0.587	1.825	0.541	-	-	-	
*Indigofera cordifolia*	0.563	0.318	0.536	0.641	-	-	-	[16]
*Leucaena leucocephala*	-	-	-	-	5	2.1	0.8	
*Indigofera oblongifolia*	0.683	0.458	7.284	0.781	-	-	-	[51]
*Ipomoea pes-caprae*	1.491	0.691	3.849	1.321	-	-	-	
*Suaeda fruticose*	-	-	-	-	32	-	1.5	[9]
*Launaea resedifolia*	0.899	0.557	1.723	1.621	-	-	-	
*Leucas urticifolia*	0.302	0.577	1.011	0.841	-	-	-	
*Prosopis cineraria*	1.113	0.224	1.324	0.361	-	-	-	[16]
*Prosopis glandulosa*	0.755	0.356	1.693	0.721	-	-	-	
*Prosopis juliflora*	0.579	0.282	2.069	0.481	-	-	-	
*Salsola imbricata*	0.147	0.197	0.732	1.211	6	-	-	[52]
*Salvadora oleoides*	0.164	0.481	0.726	1.381	-	-	-	
*Thespesia populnea*	0.305	0.256	8.684	1.511	-	-	-	
*Zaleya pentandra*	0.253	1.152	2.268	0.661	-	-	-	

**Table 3:** Effect of tannins on rumen methane production on animals.

Plants	Ruminant	Level of Inclusion (g kg^−1^)	Decline in CH_4_ Content	Effect on Other Parameters	References
*Acacia mearnsii*	Sheep	41	9.90%	23% reduction in tannin and 20% in monensin	[53]
	Cattle	9	31%	Digestibility reduced	
*H. coronarium*	Cows	Sole feed	2.35%	-	[54]
*Lespedeza cuneata*	Goats	Sole feed	51.40%	Digestibility and protozoa numbers decreased Total volatile fatty acid unaffected	[55]
*Quebracho tannins*	Beef cattle	10–20	No effect	No effect on digestibility Total volatile fatty acid decreased	
*Lotus pedunculatus*	Sheep	As sole feed	No effect	No effect	[56]
*Lespedeza striata*	Goats	As sole feed	32.9–58.4%	Digestibility and protozoal numbers decreased Total volatile fatty acid unaffected	[57]

### 3.3. Alkaloids and Glucosinolates

Alkaloids are secondary compounds present in 20% of plant species. Many cases of poisoning in cattle are reported in Europe. *T. baccata* contains highly toxic taxine alkaloids which cause deathblow because of heart attack [58]. *D. stramonium* contains tropane alkaloids which are responsible for convulsions, delirium, pupil dilation, dry mouth, and coma in cattle [59]. *C. autumnale* have colchicine comprising 70% of the total alkaloid content and causing multi-organ failure. Therefore, the exclusion of noxious plants from pastures is highly recommended [28]. Glucosinolates are S-containing secondary compounds found in almost all species of Brassica. The maximum glucosinolate tolerance level in steers, calves, cows, goats, and sheep are 15, 7.7, 11, 16, and 2 µmol per gram diet [60]. Glucosinolates are biologically inactive molecules but products from glucosinolates degradation are biologically active and have very differentiated effects such as goitrogenecity, mutagenicity, hepatotoxicity, and nephrotoxicity. Further biological effects of glucosinolates on ruminants are described in Table 4 [50].

### 3.4. Voluntary Feed Intake (Fiber and Salt)

Intake in ruminants is influenced by hunger. Ruminants select their diet by using mouth and nose receptors. Feed having imbalanced nutrients and toxins limits the food consumption. Voluntary feed intake is controlled by the relationship between the clearance of DM in the rumen and the quantity of beneficial energy to the animal. Indigestibility of fiber limits the feed intake for halophytic meadows, so in order to improve digestible organic matter voluntary feed intake should be increased [15]. Intake potential depends on the availability, quantity, and spatial distribution of herbage. Forage bushes have a more spatial distribution of edible nutrients. In sheep grazing pasture when DM falls below 3 t ha^−1^, the prospective ingestion also declines. Positive and negative effects of salt on voluntary feed intake depend on salt concentrations in feed [61]. As salt ingestion rises, water consumption also rises, which excretes slightly digested biomass across the animal gut. Ruminants are partially capable of salt ingestion, absorption, and excretion. More than 5% amount of NaCl and KCl in fodder/water decreases feed intake and changes the frequency of meals [44].

### 3.5. Toxins

Voluntary feed intake is not only influenced by a high quantity of fiber or salt but also by the toxic compounds present in the feed. Rates of detoxification are facilitated by the rate of toxin intake by the animal. All these processes require energy, water, and protein. Plants grown in inappropriate environments accumulate secondary compounds having anti-nutrient characteristics with adverse effects on feeding and livestock productivity [16]. Some of them are listed in Table 5. Toxins cause immune-competence reduction, decrease palatability, have an adverse impact on animal growth and reproduction, reduce the digestibility of essential nutrients, cause potential weight loss, and ultimately lead to animal morbidity and mortality [25].

### 3.6. Relative Palatability

**Figure 3:**
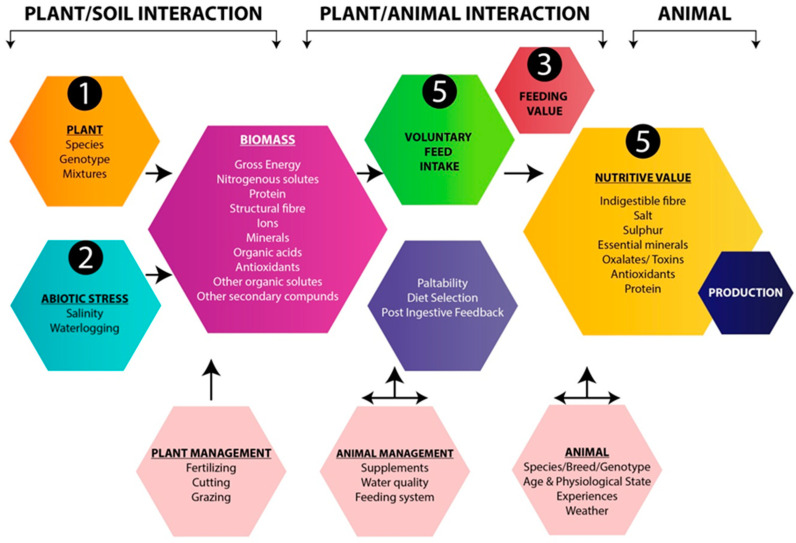
Role of relative palatability of animal for plant fodder dietary intake.

The ratio between the quantity of feed consumed by animals and the quantity offered for a certain period of time is known as palatability. The presence of high salt content in halophytic plants is a foremost restraint to their palatability. Relative palatability varies among all the species of halophytes. Palatability depends on the relative abundance of the species under rangeland and animal (species, age, health, and dietetic status) [22] (Figure 3). Table 6 shows the palatability of different halophytes by different animals.

## 4. Biomass Production and Growth Potential of Halophytes under Saline Water or Saline Soils

Halophytes can grow in saline to extremely saline habitats and have particular characteristics which enable them to tolerate salinity by various eco-physiological mechanisms. These plants are naturally grown or cultivated in salt-affected lands such as in saline semi-deserts, swamps, marshes, degraded soils, and seashores. Many of the halophytic plant species and salt-tolerant fodder species provide a valuable reserve feed for grazing animals, particularly under drought conditions or to fill regular gaps in feed supply caused by seasonal conditions [70]. The value of certain halophytic shrubs, legumes, and grass species has been recognized by their incorporation in pasture improvement programs in many salt-affected regions throughout the world [71]. There have been recent advances in selecting species with high biomass and protein levels and the ability to survive a wide range of environmental conditions including salinity. Twenty-five (25) t ha^−1^ of *A. lentiformis*, 17 t ha^−1^ of *A. nummularia,* and 15 t ha^−1^ of *A. halimus* yield were collected for differential industrial output at 20 dS m^−1^. Some grass species, such as *P. stricta*, tall wheat grass (*T. ponticum*), and a mixture of clover (*T. michelianum*) and Italian ryegrass (*L. multiflorum*) cultivated under moderate to high salinity conditions yielded 12, 5, and 2 t ha^−1^, respectively [72]. The upregulation of salt resistance mechanism in halophytes alters biomass production and cause growth loses [73]. Some other halophytes such as *H. elegans*, *T. hirsute*, *Tamarix* sp., *N. retusa*, *Salsola* sp., *A. cyanophylla,* and *Kochia* sp. have low edible DM yields and cannot support significant animal production [74]. Growing a combination of salt-tolerant grasses such as *Guinea* grass, Green panic, Pearl millet, Sorghum, and Sudan grass with legumes such as *S. sesban*, *Sesban* sp., *C. cajan,* and some *Atriplex* species would improve the feeding value of dietary rations and animal production on saline lands [75]. Numerous salt marsh plant species can be used as fodder crops under saline conditions of semi-arid and arid regions. Economic studies have indicated that farmers are making money from saline wasteland. The extension of halophytes and other salt-tolerant plants into farming practice will depend on their compatibility with the current land use system. It depends also on farmer acceptance and on the provision of adequate incentives to encourage pasture and forage crop production.

## 5. Approaches for Feeding Value Improvement of Halophytes

Numerous strategies are used to increase the feeding value. Low metabolizable energy, mineral imbalance, and toxins are constrictions on animal production. There are mainly three strategies used to improve feeding value and profitability without reducing biomass. The first is to identify new naturally salt tolerant plant species having a higher feeding value [48]. Dear and Ewing [76] conducted a major project to find a forage that can survive in salted land among all the *Melilotus* sp. They found that *M. siculus* had excellent salt tolerance among all *Melilotus* sp., and also enhanced soil fertility through N fixation. *M. siculus* had extraordinary root aeration properties. *M. siculus* had 10–10.5 MJ kg^−1^ DM nutritive value with metabolic energy and had 12% ash content [22]. New cell lines of *S. medicae* were reported by [77] that sustain efficiently in salty soil. Second, identify accessions within existing plant species having higher feeding value. Norman et al. [23] tried to increase the feeding value of *A. nummularia* by screening natural variation. In the project, this was the first effort to detect plants with greater nutritive value. Metabolizable energy, relative palatability, biomass production, amount of condensed protein, and S were the key factors for the selection criteria. Metabolizable energy was recorded from 6.6–10 MJ kg^−1^ DM, while crude protein was recorded from 11–19% DM. While the project has not been completed yet, before commercial release these clones will be examined in a variety of production environments. Third, introduce genes for salt tolerance by molecular or breeding techniques into existing plants having higher feeding value. This is the most expensive strategy to implement by targeting the genes that synthesize glycine betaine. Genes associated with the positive production of osmoregulators should be encouraged to improve ruminant production to embark upon deleterious osmoregulators such as oxalate. The screening cost of nutritive value and defining palatability are the preventive factors for plant improvement activities [1].

## 6. Effect of Halophytic Fodder on Animal Performances

### 6.1. Animal Meat Quality and Halophytic Fodder

Secondary compounds in halophytes improved animal growth performance resulting in higher weights and meat quality. Tannin wood extract showed a higher average daily gain, improving feed efficiency. Tannins containing feed increased linoleic acid without disturbing vaccenic acid [78]. Effect of tannins in ruminant meal productivity and quality summaries are provided in Table 7.

Supplementation of condensed rich forages decreased weights by 2.08 kg in goats and 0.4 kg in sheep which is 9% and 1% of their primary weights, respectively. *Leucaena* increased weight gain in ruminants due to containing tannin and saponin [51]. Riley [47] studied the growth rate of lambs by feeding them on *T. barclayana*, *S. esteroa,* and *S. bigelovii* straw. Carcass merit was excellent in all lambs. Performance of Sindhi calves was measured on various concentrations of A. nummularia (15–60%). They found highest crude protein (77%) and DM (75%) at 15% A. nummularia, while 95% carbohydrates by feeding on 30% A. nummularia. *S. bigelovii* forage effects positively the growth of goat kids and milk production. El-Shaer, [26] reported that sheep lost 26 g day^−1^ in spring season as compared to summer season (134 g day^−1^). Although pasture reached its best condition, ruminants were incapable of keeping their weights. Halophytes covered only 35% of goat energy requirements. Rams lost weight 48 g day^−1^ in drought season but increased weight by 24 g day^−1^ in browsing season by feeding A. nummularia, while in bucks increased weight occurred in drought (22.8 g day^−1^) as well as in browsing seasons (98.1 g day^−1^). Sun et al. [84] examined the quality of meat and fatty acid profile of tissue in lamb by feeding different concentration of *S. glauca* seeds. Results summaries are provided in Table 8.

### 6.2. Wool Production

Approximately 10–14% of wool production increased after 30 to 35 g CT kg^−1^ DM consumption of *L. corniculatus* because of the high absorption of S-containing amino acids in the ruminant intestine [67].

### 6.3. Milk Production and Quality

*Leucaena* sp. (100 g day^−1^) feeding significantly increases milk production in cattle and sheep. Flavonoids change the quantity of *M. elsdenii* to upturn the production of milk in animals [79]. Tannin-containing feed improves the growth in milk quantity with better composition and enhanced fertility in ruminants [66]. The effects of tannin on milk production and quality of ruminants are described in Table 9. Al Suwaiegh et al. [86] recorded higher milk yields in dairy cows by consuming condensed tannins containing feed.

## 7. Nutritional Management and Better Use of Agro-Industrial Byproducts

Rural populations depend on livestock, including mainly goats or sheep and their byproducts. Agricultural farming is limited due to short or uncertain rains, shortages of irrigated water, and salinity. Animal feeding is a severe problem due to the continuous degradation of rangeland which leads to economic instability by increasing feedstuff prices globally [95]. Moreover, climate change extends the drought period which makes this situation more complicated. Grazing of livestock on ruined lands with inferior fodder quality affects their production [96]. Essence fodders such as barley and corn are usually used, but it is very expensive, and their impact on livestock performances is unsatisfactory. Some cost-effective agro-industrial by-products are proven effective to improve animal performance [97]. Byproducts are produced during the production of core products and are referred to as agro-industrial by-products (molasses, tomato, and fruit pulps). They are cost-effective and less fibrous with a great nutritional profile. In many countries, agro-industrial byproducts are produced in large quantities, but their utilization is still limited due to the proximity for storing and transport of the agro-industrial by-products to animal flocks. New technologies have to be developed to overcome this situation [98].

### 7.1. Agro-Industrial By-Products Ensiling and Feed Blocks

Ensiling the specific food industry byproducts is an effective practice for healthier usage of agro-industrial by-products in livestock. In some Mediterranean farms, ensiled citrus or tomato pulp with olive lump are used in ruminant diets [99]. Feed blocks are the jumble of agro-industrial by-products such as salt, urea, and molasses mixed with water. In Australia and Ethiopia, for rheostat parasites therapeutic blocks containing anthelmintic agents are used [100]. Mineral-enriched feed blocks help to alleviate mineral scarcity and increase animal reproduction. Feed blocks can switch essence forages and alleviate costs without compromising on animal performances [101] (Table 10).

### 7.2. Agro-Industrial Byproducts-Based Pellets

Another promising option is to conserve agro-industrial by-products by forming them into pellets. Rudiger et al. [105] developed olive cake-based pellets by using olive, wheat, rapeseed, wheat flour, salt, and minerals. Urea was separated to evade toxification with extreme ammonia in the animal rumen. Pellets can be consumed in higher quantities due to their small size. Sheep consumed these pellets by around 3 kg day^−1^ and the price was around half of the price of imported and subsidized *lucerne* pellets [106]. Agro-industrial byproducts-based pellets can thus satisfy the feed demands of farmers.

### 7.3. Role of Molasses and Other Amendments Mixture with Halophytic Fodder to Increase Animal Palatability

Mostly halophytes have an adequate quantity of protein which is not enough to fulfill the N supplies of ruminants, along with large fiber and ash content which limits their consumption and digestibility. For better utilization and efficient digestion, energy sources should be supplemented to animals [107]. Molasses can be used in animal feed at rates of even more than 70% DM. Molasses balances the availability of nutrients in metabolism by supplying bypass nutrients. Molasses optimizes rumen fermentation by providing fermentable urea [39]. Molasses stimulates rumen fermentation and acts as a vehicle for urea and minerals. The incorporation of urea with molasses-based pellets is a very effective technology for small village farmers. Molasses can be used in the original state to reduce processing costs. Molasses increases the weight gain of fattening steers in Cuba [108]. In Columbia, steers gain 800 g day^−1^ live weight by feeding melote with 2.5% urea. Researchers found 1 kg day^−1^ weight gain of bulls in Cuba on a mixture of ad Libitum molasses plus urea with fish meal and they found 56 g day^−1^ weight gain in lambs fed on molasses with urea-treated straw [109]. Molasses also causes toxicity, affects animal eyesight potentially also damaging their brains, and leads to necrosis in animals. However, no toxicity has been reported when molasses is used with high-protein forages such as sweet potato leaves. There is no commercial application of molasses feeding due to management difficulties caused by the viscous nature of molasses [110]. On feeding berseem hay, sheep gain 71 g or goats gain 65 g daily while feeding on *H. strobilaceum,* and broiler litter silage sheep gain 73 g or goats gain 71 g daily. Results showed that feeding on silage (E£ 1.2) is more economical than the conventional diet (E£ 3.7) [12].

Alsersy et al. [111] concluded that a mixture of saltbush with barley significantly increases feed intake in sheep and improves animal growth. When feeding solely on *A. nummularia,* sheep showed very poor performance, while feeding on Rhodes grass had the utmost weight gain. The mixture of halophytic turfs, pulses, and bushes maximizes the palatability of animals. In the *Acacia cyanophylla* foliage, polyethylene glycol improves performance (palatability, weight increase, and wool growth) in ruminants [112]. Attia-Ismail, [113] reduced saponin and tannins by an ensiling process and increased animal palatability by feeding them on the ensiling mixture. About 15% of body weight was increased in lambs by using monensin [114]. A mixture of *A. nummularia* with barley (50%) and date seeds (50%) are an excellent cradle of energy. Supplementation of 50% barley grains with *A. nummularia, Acacia saligna,* and *A. semibaccata* increased digestion coefficients in ruminants [115]. Cattle palatability increased by adding oat leaves to their feed. A mixture of halophytic shrubs with *Brassica* meal increases milk production in ruminants [116].

## 8. Constraints on Halophyte Consumption

Ash content, lignification, plant secondary metabolites, and non-protein N content are the constraints of low palatability and consumption of some halophytes in animals.

### 8.1. Ash Content and Lignification Factor

Some halophytes have high ash content: mainly, Ca, silica, K, and Na. Sheep can tolerate only 100–150 g day^−1^ of sodium chloride in feed. High Na and K content decreases digestibility by curbing rumen turnover times which limits feed intake. *Atriplex* species have high Na contents of mainly more than 7% DM [11]. Hence, a mixture of saltbushes with low salt fodder is required. Providing fresh drinking water with saltbushes would reduce salt stress and improve feed consumption [117]. Halophytic plants have contained high fiber concentration, cellulose, and hemicellulose which reduces the digestibility of the nutrients. The poor intake of these halophytic plants is due to the degree of cell wall constituent digestion. Within halophytic species, there is a negative correlation between forage lignin content and nutrient utilization [118]. The contents of the cell wall and cytoplasm as well as the cell wall structure are the factors that affect voluntary forage intake and utilization. Forage with high fiber content is mainly selected by cattle rather than sheep and goats [6].

### 8.2. Plant Secondary Metabolites

Plant secondary metabolites hamper feed intake and nutrient availability. Plant secondary metabolites inhibit those microbes and fungi, which are very defensive to animals. High levels of tannins (> 60 g kg^−1^ DM) decrease the palatability of feeds and inhibit rumen microbial fermentation and abomasal or intestinal function [28]. More than 4000 mg N kg^−1^ DM in the animal regime causes anoxia by converting hemoglobin to methaemoglobin in the rumen. About 8000 mg N kg^−1^ DM reduced 60% of feed intake in sheep [68]. Oxalates precipitate insoluble calcium oxalate present in the kidneys and cause kidney failure and ultimate death. Lectins are the reasons for red blood agglutination by binding carbohydrate-containing molecules which interfere with nutrient absorption and cause diarrhea [5]. Certain halophytes contain a reasonable amount of condense protein which is enough to fulfil the N requirement of grazing animals. This N is not fully used by the animals due to carrying 50% non-protein N. A sufficient energy source would be required for their metabolism [52].

### 8.3. Alleviation of the Undesirable Secondary Compounds in Fodder

All these factors might be improved by specific treatments. Physical treatments (cutting, sopping, and drying), chemical treatment (Polyethylene glycol), and ensiling can be used in biological treatments. In a water soaking process, seeds soak in water for 6 h and then dry at 60 °C to improve feed intake [119]. Water detoxified removal processes also improved ruminant performance, but formaldehyde treatment has no influence on the production of milk. To enhance the utilization of halophytic fodders, use a jumble of halophytic grasses, legumes, and shrubs which exploits the feeding value of the fodder, as that could be a suitable way out [120].

## 9. Concluding Remarks and Future Prospective

In developing countries, feeding demands impose pressure on animal production enterprises. To increase the nutritive status of livestock, utilization of marginal resources is necessary for producing feed for animals. Halophytic forage yields large biomasses in saline land where non-halophytes cannot even cultivate. A wide range of halophytes are used to improve ruminant heath, performance, and meat quality. Halophytes are a potential source of nutrients for ruminants, but energy supplements are also required to overcome nutrient requirements. Some anti-nutritive factors restrict the utilization of halophytes in livestock feeding. So proper mixing of these species with other abetments dilutes harmful effects of this factor and improves animal performance. To meet the food demands of a growing population, strategies are required for high ruminant productivity. Production of livestock is the foremost income cradle of farmers. Due to seasonal rainfall, these areas have low fodder potential. During food scarcity periods, ruminants living in these regions are defied by nutritive scarcity, which influences their productivity and performance. To expand the livestock production system, we should develop inventive expertise pointing to the intensification of forage, improve diet quality, cost-effectiveness, and proper control of livestock watering. Awareness should be created for rural farmers to optimize benefits from livestock production.

## Figures and Tables

**Table 4:** Effects of different glucosinolate levels on ruminants [50].

Ruminant	Thiocyanate Glucosinolate (mol g^−1^ Diet)	Effect on Ruminants
Calves	2.5	No effect one thyroid function and liver
Steers	14	No effect on growth and feed renovation
Cows	12	Prompted iodine deficit
	25	Feed intake and milk production decrease
	≥24	
	32	Thyroid disruption and fertility reduction
Sheep	2.3	Weight reduction
	16	Growth Reduction
	18	Thyroid weight increment
	34	Growth reduction
	<4	No effect
	≥4	Prompted iodine deficit and affected thyroid weight and histology
	1.7	Reduction in estradiol causing reproductive disorders

**Table 5:** Toxin levels in some *Acacia* species grown under saline arid areas.

Halophytes	Plant Part	Toxins	References
*A. aneura*	Phyllode	Oxalate	[43]
		Tannin	
*A. burrowii*	Flowers	Hydrogen cyanide	
*A. cambagei*	Phyllode	Hydrogen cyanide	
	Timber, Bark	Oxalate	
*A. decora*	Browse	Abortive agent	[62]
*A. deanei*	Browse	Hydrogen cyanide	
*A. cana*	Browse	Selenium	
*A. doratoxylon*	Browse	Cyanogenic glycoside	[63]
*A. longifolia*	Browse	Hydrogen cyanide	
*A. georgina*	Seeds, Pods	Fluoroacetate	[64]
	Browse	Hyrolytic enzyme only	

**Table 6:** Palatability of halophytic plants for animal fodder.

Halophytes	Goats	Camels	Sheep	References
*Acacia albida*	PP	HP	PP	[15]
*A. elbaica*	PP	HP	PP	
*A. mellifera*	FP	HP	PP	
*A. reddiana*	PP	HP	FP	[27]
*Acacia tortilis*	FP	HP	NP	
*Arocnemom glaucum*	--	HP	NP	[65]
*Astragalus eremophilus*	--	HP	NP	
*Avicennia marina*	HP	HP	NP	
*Blepharic ciliaris*	--	HP	HP	[16]
*Cadaba farinose*	PP	HP	HP	
*Cadaba oblonifia*	HP	PP	HP	[27]
*Calligonum comosum*	HP	HP	NP	[15]
*Convolvulus hvstrix*	PP	HP	HP	[48]
*Halopeplis prefaliala*	HP	HP	HP	
*Heliotropium leuteum*	HP	HP	HP	[27]
*Indigofera spinosa*	PP	HP	NP	
*Leptadenia pyrotechnica*	HP	FP	PP	
*Lycium shawii*	HP	HP	PP	[50]
*Maerua crassifolia*	HP	HP	HP	
*Ochradenus baccatus*	HP	HP	HP	[66]
*Panicum turgidum*	HP	HP	HP	
*Pergularia tomentosa*	HP	HP	FP	
*Plantago ciliate*	FP	HP	HP	[67]
*Salsola baryosma*	FP	HP	FP	[68]
*Leptadenia pyrotechnica*	FP	PP	--	
*Suaeda monaica*	HP	HP	PP	[69]
*Taverniera aegyptiaca*	FP	FP	HP	[16]
*Trichodesma ehrenbergu*	HP	HP	--	
*Zygophyllum coccineum*	PP	HP	HP	

HP: Highly palatable, PP: Poorly palatable, FP: Fairly palatable, NP: Not palatable.

**Table 7:** Tannins containing feed effects on meat quality of ruminant.

Tannin Source	Dosage (DM CT)	Period (Days)	Effects	References
*Hedysarum coronarium*	1.8%	63	Increase linoleic acid	[79]
*Ceratonia siliqua*	2.7%	45	Increase rumenic acid and linoleic acid, Reduce n-3 FA	[80]
*Sorghum bicolor*	1.7–3.5%	103–123	No effect in muscle FA Composition	[81]
*S. quebracho*	4.0%	60	Increase t10-18:1, total trans-18:1 and PUFA Reduce SFA	[82]
*Acacia mearnsii*	14.1%	260–283	Reduce rumenic acid	[83]
*Juniperus pinchotii*	3.1–4.4%	86	Increase SFA, rumenic acid and Δ-9 Desaturase index	
*Terminalia chebula*	0.6–1.8%	90	Increase rumenic acid, MUFA and linoleic acid	

**Table 8:** Effect of *S. glauca* seed on lamb meat quality and fatty acid composition [85].

Treatments	Control	T_1_	T_2_	T_3_
Slaughter weight (kg)	28	29	31	30
Carcass weight (kg)	10	11.5	11	12
Dressing (%)	38.80	40.29	38.02	38.89
pH (24 h)	5.75	5.60	5.55	5.65
C10:0	0.12	0.16	0.17	0.11
C12:0	0.22	0.27	0.43	0.20
C14:0	3.06	3.68	4.75	2.83
C16:0	21.59	24.96	25.46	23.13
C16:1	1.08	1.37	1.23	1.00
C18:0	19.95	21.00	20.73	24.90
C18:1 cis-9	33.62	34.22	30.47	30.48
C18:1 trans-11	1.16	1.63	1.69	2.24
C18:2	0.29	0.40	0.30	0.44
C18:2 n−6	8.78	6.25	7.95	8.34
C18:3	0.03	0.03	0.04	0.04
C18:3	0.43	0.49	0.51	0.52
C20:0	0.15	0.13	0.19	0.17
C20:1	0.04	0.04	0.06	0.06
C20:3	0.28	0.17	0.18	0.19
C20:4	4.56	2.40	2.93	2.50
C22:6 n−3	0.14	0.10	0.10	0.10
Other fatty acid	4.47	2.66	2.69	2.71
PUFA	14.42	9.88	12.11	12.17
SFA	46.86	50.19	51.67	51.34
P:S	0.30	0.19	0.20	0.23
n-6	13.66	8.86	11.10	11.07
n-3	0.55	0.61	0.62	0.63
n-6/n-3 ratio	14.6	18.5	17.5	1.72

Control: 300 g concentrates with ad libitum *L. chinensis* hay; T_1_: 300 g concentrates +150 g *S. glauca* seed with ad libitum *L. chinensis* hay; T_2_: 300 g concentrates +300 g *S. glauca* seed with ad libitum *L. chinensis* hay; T_3_: 300 g concentrates +450 g *S. glauca* seed with ad libitum *L. chinensis* hay.

**Table 9:** Dietary tannin effects on ruminant milk.

Tannin Source	Dosage (DM CT)	Duration (Days)	Effects	References
*Schinopsis* *quebracho-colorado*	70%	27	Fatty acids profile of milk remains the same	[87]
*Hedysarum coronarium*	2.7%	56	Increases linoleic acid and milk fat content, as well as a reduction in vaccenic acid, rumenic acid, and milk urea content	[88]
*Lens culinaris*	74%	50	Reduction in linoleic acid, oleic and stearic acids, and milk fat content	[89]
*Olea europaea*	94%	50	Reduction in linoleic, oleic, and stearic acids, as well as energy-corrected milk yield	[90]
*S. balansae*	3%	21	Reduce milk urea content Increase linoleic acid	[91]
*F. esculentum*	82%	26	Increase linoleic acid Reduce vaccenic acid and t10-18:1	[92]
*C. sativa*	50%	30	Fatty acids profile of milk remains the same	[93]
*S. quebracho*	2%	30		[69]
*A. mearnsii*	400 g day^−1^CT	25		[94]

**Table 10:** Effect of feed blocks on the performance of lambs related to the growth rate and cost reduction.

Basal Diet	Supplements	Growth Rate (g Day ^−1^)	Cost Reduction	References
Stubble browsing	Conc. (250 g day^−1^)	96	−81%	[49]
	Conc. (150 g day^−1^), wheat bran (10%), olive cake (40%), poultry litter (25%), bentonite (20%), salt (5%)	137	−80%	
Wheat straw	Conc. (500 g day^−1^)	64	−81%	[102]
	Conc. (125 g day^−1^) + Wheat bran (25%), wheat flour (15%), olive cake (30%), rapeseed meal (10%), urea (4%), quicklime (8%), salt (5%), minerals (1%)	67	−12%	
Straw (310 g day^−1^)	Conc. (800 g day^−1^)	121	−11%	[47]
	Conc. (300 g day^−1^) + Wheat bran (28%), barley (10%), molasses (44%), sesames hull (5%), white cement (5%), minerals (3%), urea (5%)	110	−11%	
Fresh *Acacia* leaves	Wheat bran (28%), olive cake (38%), wheat flour (11%), quicklime (12%), salt (5%), minerals (1%), urea (5%)	15	−11%	[103]
	Wheat bran (23%), olive cake (31.2%), wheat flour (9%), quicklime (9.9%), salt (4.1%), minerals (0.8%), urea (4.1%), PEG (18%)	62	−11%	
Rangeland browsing	Conc. (300 g day^−1^)	26	−11%	[104]
	Wheat bran (28%), olive cake (38%), wheat flour (11%), quicklime (12%), salt (5%), minerals (1%), urea (5%)	40	−11%	

## Data Availability

Not applicable.

## References

[1] Munir N., Hasnain M., Roessner U., Abideen Z. (2021). Strategies in Improving Plant Salinity Resistance and Use of Salinity Resistant Plants for Economic Sustainability. Crit. Rev. Environ. Sci. Technol..

[2] Hasnain M., Abideen Z., Anthony Dias D., Naz S., Munir N. (2022). Utilization of Saline Water Enhances Lipid Accumulation in Green Microalgae for the Sustainable Production of Biodiesel. BioEnergy Res..

[3] Tyagi J., Ahmad S., Malik M. (2022). Nitrogenous Fertilizers: Impact on Environment Sustainability, Mitigation Strategies, and Challenges. Int. J. Environ. Sci. Technol..

[4] Kakarla R., Choi J.-W., Yun J.-H., Kim B.-H., Heo J., Lee S., Cho D.-H., Ramanan R., Kim H.-S. (2018). Application of High-Salinity Stress for Enhancing the Lipid Productivity of *Chlorella sorokiniana* HS1 in a Two-Phase Process. J. Microbiol..

[5] Saha B., Chowardhara B., Awasthi J.P., Panda S.K., Panigrahi K.C.S., Shahnawaz M. (2021). Salinity Stress and Plant Secondary Metabolite Enhancement: In An Overview. Biotechnological Approaches to Enhance Plant Secondary Metabolites.

[6] Calone R., Bregaglio S., Sanoubar R., Noli E., Lambertini C., Barbanti L. (2021). Physiological Adaptation to Water Salinity in Six Wild Halophytes Suitable for Mediterranean Agriculture. Plants.

[7] Tıpırdamaz R., Karakas S., Dikilitas M. (2020). Halophytes and the Future of Agriculture. Handbook of Halophytes: From Molecules to Ecosystems towards Biosaline Agriculture.

[8] Turcios A.E., Cayenne A., Uellendahl H., Papenbrock J. (2021). Halophyte Plants and Their Residues as Feedstock for Biogas Production—Chances and Challenges. Appl. Sci..

[9] Saleem H., Khurshid U., Sarfraz M., Tousif M.I., Alamri A., Anwar S., Alamri A., Ahmad I., Abdallah H.H., Mahomoodally F.M. (2021). A Comprehensive Phytochemical, Biological, Toxicological and Molecular Docking Evaluation of *Suaeda fruticosa* (L.) Forssk.: An Edible Halophyte Medicinal Plant. Food Chem. Toxicol..

[10] Scopel B.R., de Miranda P.H.O., da Silva R.S., de Oliveira Alves J.V., de Veras B.O., Amorim L.C., da Rosa M.M., de Souza barbosa J.I., de Melo M.R.C.S., de Souza Bezerra R. (2021). Biological Activities and Chemical Profile from *Batis maritima* (Bataceae), a Halophyte Species with Bioprospecting Potential. Res. Soc. Dev..

[11] Attia-Ismail S.A., Loiola Edvan R., Rocha Bezerra L. (2018). Halophytes as Forages. New Perspectives in Forage Crops.

[12] Badri M., Ludidi N., Badri M., Luddi N. (2020). Halophytes as a Resource for Livestock in Africa: Present Status and Prospects: Present Status and Prospects. Handbook of Halophytes: From Molecules to Ecosystems towards Biosaline Agriculture.

[13] Elahi M.Y., Khusro A., Elghnadour M.M.Y., Salem A.Z.M., López S. (2019). Ruminal Fermentation Kinetics of Nine Halophytic Tree Species at Different Growth Stages. Agrofor. Syst..

[14] Friha M., Hamdi H., Ayeb N., Hajlaoui A., Durand D., Majdoub-Mathlouthi L. (2022). Potential Use of Natural Saline Pasture for Grazing Lambs: Effect on Digestibility, Growth Performances, Carcass and Meat Quality. Small Rumin. Res..

[15] Attia-Ismail S.A., El Shaer H.M., Squires V.R. (2016). Nutritional and Feed Value of Halophytes and Salt Tolerant Plants. Halophytic Salt-Tolerant Feed.

[16] Attia-Ismail S.A., Badri M., Luddi N. (2020). Nutrient Feasibility of Halophytic Feed Plants. Handbook of Halophytes: From Molecules to Ecosystems towards Biosaline Agriculture.

[17] Rahman M.M., Mostofa M.G., Keya S.S., Siddiqui M.N., Ansary M.M.U., Das A.K., Rahman M.A., Tran L.S.-P. (2021). Adaptive Mechanisms of Halophytes and Their Potential in Improving Salinity Tolerance in Plants. Int. J. Mol. Sci..

[18] Atia A., Debez A., Rabhi M., Barhoumi Z., Haouari C.C., Gouia H., Abdelly C., Smaoui A. (2019). Salt Tolerance and Potential Uses for Saline Agriculture of Halophytes from the Poaceae. Sabkha Ecosystems.

[19] Dawei Z., Vu T.S., Huang J., Chi C.Y., Xing Y., Fu D.D., Yuan Z.N. (2019). Effects of Calcium on Germination and Seedling Growth in *Melilotus officinalis* L. (Fabaceae) under Salt Stress. Pak. J. Bot.

[20] Mausz M.A., Airs R.L., Dixon J.L., Widdicombe C.E., Tarran G.A., Polimene L., Dashfield S., Beale R., Scanlan D.J., Chen Y. (2022). Microbial uptake dynamics of choline and glycine betaine in coastal seawater. Limnol. Oceanogr..

[21] Abdurzakova A.S., Astamirova M.A.M., Magomadova R.S., Israilova S.A., Khanaeva K.R., Khasueva B.A., Umaeva A.M., Shakhgirieva Z.I. (2019). Halophytes of Tersko-Kumsk Lowland Area, Their Protection and Rational Use. KnE Life Sci..

[22] Hasnain M., Munir N., Abideen Z., Zulfiqar F., Koyro H.W., El-Naggar A., Caçador I., Duarte B., Rinklebe J., Yong J.W.H. (2023). Biochar-Plant Interaction and Detoxification Strategies under Abiotic Stresses for Achieving Agricultural Resilience: A Critical Review. Ecotoxicol. Environ. Saf..

[23] Bhat R.A., Hakeem K.R., Badri M., Luddi N. (2020). Biomass Production of Various Halophytes. Handbook of Halophytes: From Molecules to Ecosystems towards Biosaline Agriculture.

[24] Centofanti T., Bañuelos G. (2019). Practical Uses of Halophytic Plants as Sources of Food and *Fodder*. Halophytes and Climate Change: Adaptive Mechanisms and Potential Uses.

[25] El-Shaer H.M. (2004). Potentiality of Halophytes as Animals Fodders under Arid Conditions of Egypt. Ferchichi A.(comp.), Ferchichi a.(collab.). Rehabilitation des Paturages et des Parcours en Milieu Mediterraneens.

[26] Yasin Ashraf M., Awan A.R., Anwar S., Khaliq B., Malik A., Ozturk M. (2020). Economic Utilization of Salt-Affected Wasteland for Plant Production: A Case Study from Pakistan. Handbook of Halophytes: From Molecules to Ecosystems towards Biosaline Agriculture.

[27] Wessels A.G. (2022). Influence of the gut microbiome on feed intake of farm animals. Microorganisms.

[28] Öztürk M., Altay V., Güvensen A. (2019). Sustainable Use of Halophytic Taxa as Food and Fodder: An Important Genetic Resource in Southwest Asia. Ecophysiology, Abiotic Stress Responses and Utilization of Halophytes.

[29] Stevanovic Z., Stankovic M.S., Stankovic J., Janackovic P., Stankovic M. (2019). Use of Halophytes as Medicinal Plants: Phytochemical Diversity and Biological Activity. Halophytes and Climate Change: Adaptive Mechanisms and Potential Uses.

[30] Gouda M.S., Elsebaie E.M. (2016). Glasswort (*Salicornia* spp.) as a Source of Bioactive Compounds and Its Health Benefits: A Review. Alex. J. Food. Sci. Technol.

[31] Reza Yousefi A., Rashidi S., Moradi P., Mastinu A. (2020). Germination and Seedling Growth Responses of *Zygophyllum fabago*, *Salsola kali* L. and *Atriplex canescens* to PEG-Induced Drought Stress. Environments.

[32] Lüttge U. (2019). Elimination of Salt by Recretion: Salt Glands and Gland-Supported Bladders in Recretohalophytes. Halophytes and Climate Change: Adaptive Mechanisms and Potential Uses.

[33] Li H., Xu C., Han L., Li C., Xiao B., Wang H., Yang C. (2022). Extensive Secretion of Phenolic Acids and Fatty Acids Facilitates Rhizosphere PH Regulation in Halophyte *Puccinellia tenuiflora* under Alkali Stress. Physiol. Plant..

[34] Nikalje G.C., Suprasanna P. (2018). Coping with Metal Toxicity–Cues from Halophytes. Front. Plant Sci..

[35] Liu Y., Yang M., Zheng L., Nguyen H., Ni L., Song S., Sui Y. (2020). Antioxidant Responses of Triangle Sail Mussel *Hyriopsis cumingii* Exposed to Toxic *Microcystis aeruginosa* and Thermal Stress. Sci. Total Environ..

[36] Mohammed H.A., Ali H.M., Qureshi K.A., Alsharidah M., Kandil Y.I., Said R., Mohammed S.A.A., Al-Omar M.S., Al Rugaie O., Abdellatif A.A.H. (2021). Comparative Phytochemical Profile and Biological Activity of Four Major Medicinal Halophytes from Qassim Flora. Plants.

[37] Eissa M.A. (2019). Effect of Compost and Biochar on Heavy Metals Phytostabilization by the Halophytic Plant Old Man Saltbush [*Atriplex nummularia* Lindl]. Soil Sediment Contam. Int. J..

[38] Nikalje G.C., Srivastava A.K., Pandey G.K., Suprasanna P. (2018). Halophytes in Biosaline Agriculture: Mechanism, Utilization, and Value Addition. Land Degrad. Dev..

[39] Ishtiyaq S., Kumar H., Varun M., Ogunkunle C.O., Paul M.S., Hasanuzzaman M., Prasad M.N.V. (2021). Role of Secondary Metabolites in Salt and Heavy Metal Stress Mitigation by Halophytic Plants. Handbook of Bioremediation.

[40] Kewan K.Z., Elkhouly A.A., Negm A.M., Javadi A. Feedstock Values of Some Common Fodder Halophytes in the Egyptian Desert. Proceedings of the 22nd International Water Technology Conference, IWTC22 Ismailia.

[41] Baysal I., Ekizoglu M., Ertas A., Temiz B., Agalar H.G., Yabanoglu-Ciftci S., Temel H., Ucar G., Turkmenoglu F.P. (2021). Identification of Phenolic Compounds by LC-MS/MS and Evaluation of Bioactive Properties of Two Edible Halophytes: *Limonium effusum* and *L. dinuatum*. Molecules.

[42] Lopes M., Sanches-Silva A., Castilho M., Cavaleiro C., Ramos F. (2021). Halophytes as Source of Bioactive Phenolic Compounds and Their Potential Applications. Crit. Rev. Food Sci. Nutr..

[43] Ebrahim H., Negussie F. (2020). Effect of Secondary Compounds on Nutrients Utilization and Productivity of Ruminant Animals: A Review. J. Agric. Sci. Pr..

[44] Attia-Ismail S.A. (2015). Plant Secondary Metabolites: Deleterious Effects, Remediation. Plants, Pollutants and Remediation.

[45] El Shaer H.M. (2010). Halophytes and Salt-Tolerant Plants as Potential Forage for Ruminants in the Near East Region. Small Rumin. Res..

[46] Giger-Reverdin S., Domange C., Broudiscou L.P., Sauvant D., Berthelot V. (2020). Rumen Function in Goats, an Example of Adaptive Capacity. J. Dairy Res..

[47] Riley J.J. (2015). Review of Halophyte Feeding Trials with Ruminants. Halophytic Salt-Tolerant Feed.

[48] Nefzaoui A., Salem H.B., El Mourid M. (2012). Innovations in Small Ruminants Feeding Systems in Arid Mediterranean Areas. New Trends for Innovation in the Mediterranean Animal Production.

[49] Masters D.G. (2015). Assessing the Feeding Value of Halophytes. Halophytic and Salt Tolerant Feedstuffs: Impacts on Nutrition, Physiology and Reproduction of Livestock.

[50] Salem H.B., Makkar H.P.S., Nefzaoui A. (2004). Towards Better Utilisation of Non-Conventional Feed Sources by Sheep and Goats in Some African and Asian Countries. Options Méditerranéennes Série A.

[51] Tripathi M.K., Mishra A.S. (2007). Glucosinolates in Animal Nutrition: A Review. Anim. Feed Sci. Technol..

[52] Molina-Botero I.C., Arroyave-Jaramillo J., Valencia-Salazar S., Rosales R.B., Aguilar-Pérez C.F., Burgos A.A., Arango J., Ku-Vera J.C. (2019). Effects of Tannins and Saponins Contained in Foliage of *Gliricidia Sepium* and Pods of *Enterolobium cyclocarpum* on Fermentation, Methane Emissions and Rumen Microbial Population in Crossbred Heifers. Anim. Feed Sci. Technol..

[53] Faustino M.V., Faustino M.A.F., Pinto D.C.G.A. (2019). Halophytic Grasses, a New Source of Nutraceuticals? A Review on Their Secondary Metabolites and Biological Activities. Int. J. Mol. Sci..

[54] Junior F.P., Cassiano E.C.O., Martins M.F., Romero L.A., Zapata D.C.V., Pinedo L.A., Marino C.T., Rodrigues P.H.M. (2017). Effect of Tannins-Rich Extract from *Acacia mearnsii* or *monensin* as Feed Additives on Ruminal Fermentation Efficiency in Cattle. Livest. Sci..

[55] Piluzza G., Sulas L., Bullitta S. (2014). Tannins in Forage Plants and Their Role in Animal Husbandry and Environmental Sustainability: A Review. Grass Forage Sci..

[56] Naumann H.D., Tedeschi L.O., Muir J.P., Lambert B.D., Kothmann M.M. (2013). Effect of Molecular Weight of Condensed Tannins from Warm-Season Perennial Legumes on Ruminal Methane Production in Vitro. Biochem. Syst. Ecol..

[57] Tavendale M.H., Meagher L.P., Pacheco D., Walker N., Attwood G.T., Sivakumaran S. (2005). Methane Production from in Vitro Rumen Incubations with *Lotus pedunculatus* and *Medicago sativa*, and Effects of Extractable Condensed Tannin Fractions on Methanogenesis. Anim. Feed Sci. Technol..

[58] Cardoso-Gutierrez E., Aranda-Aguirre E., Robles-Jimenez L.E., Castelán-Ortega O.A., Chay-Canul A.J., Foggi G., Angeles-Hernandez J.C., Vargas-Bello-Pérez E., González-Ronquillo M. (2021). Effect of Tannins from Tropical Plants on Methane Production from Ruminants: A Systematic Review. Vet. Anim. Sci..

[59] Arya S.S., Devi S., Ram K., Kumar S., Kumar N., Mann A., Kumar A., Chand G., Hasanuzzaman M., Nahar K., Öztürk M. (2019). Halophytes: The Plants of Therapeutic Medicine. Ecophysiology, Abiotic Stress Responses and Utilization of Halophytes.

[60] Amirifar A., Hemati A., Asgari Lajayer B., Pandey J., Astatkie T. (2022). Impact of Various Environmental Factors on the Biosynthesis of Alkaloids in Medicinal Plants. Environmental Challenges and Solutions.

[61] Ksouri R., Ksouri W.M., Jallali I., Debez A., Magné C., Hiroko I., Abdelly C. (2012). Medicinal Halophytes: Potent Source of Health Promoting Biomolecules with Medical, Nutraceutical and Food Applications. Crit. Rev. Biotechnol..

[62] Khan M.A., Duke N.C. (2001). Halophytes-A Resource for the Future. Wetl. Ecol. Manag..

[63] Dynes R.A., Schlink A.C. (2002). Livestock Potential of Australian Species of Acacia. Conserv. Sci. West. Aust..

[64] Cock I.E. (2011). Medicinal and Aromatic Plants-Australia.

[65] Aganga A.A., Tshwenyane S.O. (2003). Feeding Values and Anti-Nutritive Factors of Forage Tree Legumes. Pak. J. Nutr..

[66] Squires V.R., El Shaer H.M. (2015). Global Distribution and Abundance of Sources of Halophytic and Salt Tolerant Feedstuffs. Halophytic and Salt-Tolerant Feedstuffs.

[67] Nascimento T.V.C., Oliveira R.L., Menezes D.R., de Lucena A.R.F., Queiroz M.A.Á., Lima A., Ribeiro R.D.X., Bezerra L.R. (2021). Effects of Condensed Tannin-Amended Cassava Silage Blend Diets on Feeding Behavior, Digestibility, Nitrogen Balance, Milk Yield and Milk Composition in Dairy Goats. Animal.

[68] Barry T.N., McNeill D.M., McNabb W.C. Plant Secondary Compounds; Their Impact on Forage Nutritive Value and upon Animal Production. Proceedings of the XIX International Grassland Congress São Pedro.

[69] Moehlenpah A.N., Ribeiro L.P.S., Puchala R., Goetsch A.L., Beck P., Pezeshki A., Gross M.A., Holder A.L., Lalman D.L. (2021). Water and Forage Intake, Diet Digestibility, and Blood Parameters of Beef Cows and Heifers Consuming Water with Varying Concentrations of Total Dissolved Salts. J. Anim. Sci..

[70] Abd El-Hack M.E., Samak D.H., Noreldin A.E., Arif M., Yaqoob H.S., Swelum A.A. (2018). Towards Saving Freshwater: Halophytes as Unconventional Feedstuffs in Livestock Feed: A Review. Environ. Sci. Pollut. Res..

[71] Anon M. (2009). Introduction of Salt-Tolerant Forage Production Systems to Salt-Affected Lands in Sinai Peninsula in Egypt: A Pilot Demonstration Project.

[72] Masters D.G., Benes S.E., Norman H.C. (2007). Biosaline Agriculture for Forage and Livestock Production. Agric. Ecosyst. Environ..

[73] Abideen Z., Koyro H.W., Huchzermeyer B., Ahmed M.Z., Gul B., Khan M.A. (2014). Moderate salinity stimulates growth and photosynthesis of *Phragmites karka* by water relations and tissue specific ion regulation. Environ. Exp. Bot..

[74] Katoch R. (2022). Nutritional Quality of Major Forage Grasses of Himalayan Region. Nutritional Quality Management of Forages in the Himalayan Region.

[75] Dear B.S., Ewing M.A. (2008). The Search for New Pasture Plants to Achieve More Sustainable Production Systems in Southern Australia. Aust. J. Exp. Agric..

[76] Buccioni A., Pauselli M., Viti C., Minieri S., Pallara G., Roscini V., Rapaccini S., Marinucci M.T., Lupi P., Conte G. (2015). Milk Fatty Acid Composition, Rumen Microbial Population, and Animal Performances in Response to Diets Rich in Linoleic Acid Supplemented with Chestnut or Quebracho Tannins in Dairy Ewes. J. Dairy Sci..

[77] Ballard R.A., Peck D.M. (2021). Sensitivity of the Messina (*Melilotus siculus*)–*Sinorhizobium medicae* Symbiosis to Low pH. Crop Pasture Sci..

[78] Shehata M.F. (2021). Feeding Camels on Halophytic Plants and Their Effects on Meat Quality Characteristics and Products. Management and Development of Agricultural and Natural Resources in Egypt’s Desert.

[79] Bonanno A., Di Miceli G., Di Grigoli A., Frenda A.S., Tornambè G., Giambalvo D., Amato G. (2011). Effects of Feeding Green Forage of Sulla (*Hedysarum coronarium* L.) on Lamb Growth and Carcass and Meat Quality. Animal.

[80] Silanikove N., Landau S., Or D., Kababya D., Bruckental I., Nitsan Z. (2006). Analytical Approach and Effects of Condensed Tannins in Carob Pods (*Ceratonia siliqua*) on Feed Intake, Digestive and Metabolic Responses of Kids. Livest. Sci..

[81] de Matos Teixeira A., Junior G.D., Velasco F.O., de Faria Júnior W.G., Rodriguez N.M., Rodrigues J.A., McAllister T., Gonçalves L.C. (2014). Intake and Digestibility of Sorghum (*Sorghum bicolor*, L. Moench) Silages with Different Tannin Contents in Sheep. Rev. Bras. Zootec..

[82] Piñeiro-Vázquez A.T., Jiménez-Ferrer G., Alayon-Gamboa J.A., Chay-Canul A.J., Ayala-Burgos A.J., Aguilar-Pérez C.F., Ku-Vera J.C. (2018). Effects of Quebracho Tannin Extract on Intake, Digestibility, Rumen Fermentation, and Methane Production in Crossbred Heifers Fed Low-Quality Tropical Grass. Trop. Anim. Health Prod..

[83] Patra A.K. (2014). Exploring the Benefits of Feeding Tannin Containing Diets for Enhancing the Nutritional Values of Milk and Meat of Ruminants. Indian J. Anim. Health.

[84] Sun H.X., Zhong R.Z., Liu H.W., Wang M.L., Sun J.Y., Zhou D.W. (2015). Meat Quality, Fatty Acid Composition of Tissue and Gastrointestinal Content, and Antioxidant Status of Lamb Fed Seed of a Halophyte (*Suaeda glauca*). Meat Sci..

[85] Rashid M., Archimède H. (2019). Response of Lactating Blackbelly Ewes to Fed Pellets of Leucaena leucocephala Leaves with Dry Banana Fruits. Proceedings of the Caribbean Science and Innovation Meeting.

[86] AlSuwaiegh S.B., Almotham A.M., Alyousef Y.M., Mansour A.T., Al-Sagheer A.A. (2022). Influence of Functional Feed Supplements on the Milk Production Efficiency, Feed Utilization, Blood Metabolites, and Health of Holstein Cows during Mid-Lactation. Sustainability.

[87] Beck M.R., Al-Marashdeh O., Gregorini P. (2019). Low Levels of a Seaweed (*Ecklonia radiata*) Extract Alter in Vitro Fermentation Products but Not in Combination with Quebracho (*Schinopsis quebracho-colorado*) Tannins. Appl. Anim. Sci..

[88] Cabiddu A., Molle G., Decandia M., Spada S., Fiori M., Piredda G., Addis M. (2009). Responses to Condensed Tannins of Flowering Sulla (*Hedysarum coronarium* L.) Grazed by Dairy Sheep: Part 2: Effects on Milk Fatty Acid Profile. Livest. Sci..

[89] Morales R., Ungerfeld E.M. (2015). Use of Tannins to Improve Fatty Acids Profile of Meat and Milk Quality in Ruminants: A Review. Chil. J. Agric. Res..

[90] Molina-Alcaide E., Yáñez-Ruiz D.R. (2008). Potential Use of Olive By-Products in Ruminant Feeding: A Review. Anim. Feed Sci. Technol..

[91] Vera N., Gutiérrez-Gómez C., Williams P., Allende R., Fuentealba C., Ávila-Stagno J. (2022). Comparing the Effects of a Pine (*Pinus radiata* D. Don) Bark Extract with a Quebracho (*Schinopsis balansae* Engl.) Extract on Methane Production and In vitro Rumen Fermentation Parameters. Animals.

[92] Amelchanka S.L., Kreuzer M., Leiber F. (2010). Utility of Buckwheat (*Fagopyrum esculentum* Moench) as Feed: Effects of Forage and Grain on in Vitro Ruminal Fermentation and Performance of Dairy Cows. Anim. Feed Sci. Technol..

[93] Colombini S., Broderick G.A., Galasso I., Martinelli T., Rapetti L., Russo R., Reggiani R. (2014). Evaluation of *Camelina sativa* (L.) Crantz Meal as an Alternative Protein Source in Ruminant Rations. J. Sci. Food Agric..

[94] Denninger T.M., Schwarm A., Birkinshaw A., Terranova M., Dohme-Meier F., Muenger A., Eggerschwiler L., Bapst B., Wegmann S., Clauss M. (2020). Immediate Effect of *Acacia mearnsii* Tannins on Methane Emissions and Milk Fatty Acid Profiles of Dairy Cows. Anim. Feed Sci. Technol..

[95] Torkashvand N., Soltan Dalal M.M., Mousivand M., Hashemi M. (2020). Canola Meal and Tomato Pomace as Novel Substrates for Production of Thermostable *Bacillus Subtilis* T4b Xylanase with Unique Properties. Biomass Convers. Biorefin..

[96] Onte S., Bhattacharjee S., Arif M., Dey D. (2019). Non-Conventional Feed Resources. Agriallis.

[97] Mishra B., Varjani S., Karthikeya Srinivasa Varma G. (2019). Agro-Industrial by-Products in the Synthesis of Food Grade Microbial Pigments: An Eco-Friendly Alternative. Green Bio-Processes.

[98] Meral R., Kose Y.E., Ceylan Z., Cavidoglu İ. (2022). The Potential Use of Agro-Industrial by-Products as Sources of Bioactive Compounds: A Nanotechnological Approach. Stud. Nat. Prod. Chem..

[99] Mirzaei-Aghsaghali A., Maheri-Sis N. (2008). Nutritive Value of Some Agro-Industrial by-Products for Ruminants-A Review. World J. Zool.

[100] Moore R. (2018). Principles of Animal Nutrition.

[101] Meneses M., Martínez-Marín A.L., Madrid J., Martínez-Teruel A., Hernández F., Megías M.D. (2020). Ensilability, in Vitro and in Vivo Values of the Agro-Industrial by-products of Artichoke and Broccoli. Environ. Sci. Pollut. Res..

[102] Salem H.B., Znaidi I.-A. (2008). Partial Replacement of Concentrate with Tomato Pulp and Olive Cake-Based Feed Blocks as Supplements for Lambs Fed Wheat Straw. Anim. Feed Sci. Technol..

[103] Delgadillo-Puga C., Cuchillo-Hilario M. (2021). Reviewing the Benefits of Grazing/Browsing Semiarid Rangeland Feed Resources and the Transference of Bioactivity and Pro-Healthy Properties to Goat Milk and Cheese: Obesity, Insulin Resistance, Inflammation and Hepatic Steatosis Prevention. Animals.

[104] Pinto Filho J.S., Cunha M.V., Souza E.J.O., Santos M.V.F., Lira M.A., Moura J.G., Rodrigues J., Silva C.S. (2019). Performance, Carcass Features, and Non-Carcass Components of Sheep Grazed on Caatinga Rangeland Managed with Different Forage Allowances. Small Rumin. Res..

[105] Rudiger U., El Ayed M., Idoudi Z., Frija A., Nefzaoui A., Gharsi M. (2021). Nutrient-Dense Livestock Feed Pellets Manufactured in Tunisia Can Compete with Imported Feed Concentrates. Bus. Model Brief..

[106] Collado R., Monedero E., Casero-Alonso V.M., Rodríguez-Aragón L.J., Hernández J.J. (2022). Almond Shells and Exhausted Olive Cake as Fuels for Biomass Domestic Boilers: Optimization, Performance and Pollutant Emissions. Sustainability.

[107] Gihad E.A., Shaer H.M.E.L., Squires V.R., Ayoub A.T. (1994). Utilization of Halophytes by Livestock on Rangelands Problems and Prospects. Halophytes as a Resource for Livestock and for Rehabilitation of Degraded Lands.

[108] El Shaer H.M., Grigore M.N. (2021). Potential Use of Halophytes and Salt-Tolerant Forages as Animal Feed in the Arab Region: An Overview. Handbook of Halophytes.

[109] Shellito S.M., Ward M.A., Lardy G.P., Bauer M.L., Caton J.S. (2006). Effects of Concentrated Separator By-Product (Desugared Molasses) on Intake, Ruminal Fermentation, Digestion, and Microbial Efficiency in Beef Steers Fed Grass Hay. J. Anim. Sci..

[110] Preston T.R., Sansoucy R., Aarts G. Molasses as Animal Feed: An Overview; Sugarcane as Feed, FAO Animal Production and Health Papers 72. In Proceeding of the FAO Expert Consultation.

[111] Alsersy H., Salem A.Z.M., Borhami B.E., Olivares J., Gado H.M., Mariezcurrena M.D., Yacuot M.H., Kholif A.E., El-dawy M., Hernandez S.R. (2015). Effect of M Editerranean Saltbush (*Atriplex halimus*) Ensilaging with Two Developed Enzyme Cocktails on Feed Intake, Nutrient Digestibility and Ruminal Fermentation in Sheep. Anim. Sci. J..

[112] Dagar J.C., Sharma P.C., Chaudhari S.K., Jat H.S., Ahamad S. (2016). Climate Change Vis-a-Vis Saline Agriculture: Impact and Adaptation Strategies. Innovative Saline Agriculture.

[113] Attia-Ismail S.A. Factors Limiting and Methods of Improving Nutritive and Feeding Values of Halophytes in Arid, Semi-Arid and Coastal Areas. Proceedings of the International Conference on Biosaline Agriculture & High Salinity Tolerance.

[114] Polizel D.M., Marques S.S., Westphalen M.F., Gouvea V.N., de Castro Ferraz Júnior M.V., Miszura A.A., Barroso J.P.R., Limede A.C., Ferreira E.M., Pires A.V. (2020). Narasin Inclusion for Feedlot Lambs Fed a Diet Containing High Amounts of Ground Flint Corn. Sci. Agric..

[115] Selim E., Fisun K.O.C., Özdüven L., Eseceli H., Evren C., Karadağ H. (2022). In Situ and In Vitro Nutritive Value Assessment of *Styrax officinalis* L. as an Alternative Forage Source for Goat Feeding. J. Agric. Sci..

[116] Petrie J.R., Zhou X.-R., Leonforte A., McAllister J., Shrestha P., Kennedy Y., Belide S., Buzza G., Gororo N., Gao W. (2020). Development of a *Brassica napus* (Canola) Crop Containing Fish Oil-like Levels of DHA in the Seed Oil. Front. Plant Sci..

[117] Ranjbar G.H., Pirasteh-Anosheh H. (2020). Comparison of the Accumulation of Elements, Ash Content and Biomass of Some Halophytes Species under Irrigating with Sea Water. Desert Manag..

[118] EL-Saadany S.A., Omar H.H. (2018). Effect of Feeding Barki Ewesby Halophytic Plants on the Gross Chemical Composition, Elements Content and Anti-Oxidants Compounds. J. Food Dairy Sci..

[119] Abd El Tawab A.M., Khattab M.S.A. (2018). Utilization of Polyethylene Glycol and Tannase Enzyme to Reduce the Negative Effect of Tannins on Digestibility, Milk Production and Animal Performance. Asian J. Anim. Vet. Adv..

[120] Sipos P., Peles F., Brassó D.L., Béri B., Pusztahelyi T., Pócsi I., Győri Z. (2021). Physical and Chemical Methods for Reduction in Aflatoxin Content of Feed and Food. Toxins.

